# Nematode Spatial and Ecological Patterns from Tropical and Temperate Rainforests

**DOI:** 10.1371/journal.pone.0044641

**Published:** 2012-09-11

**Authors:** Dorota L. Porazinska, Robin M. Giblin-Davis, Thomas O. Powers, W. Kelley Thomas

**Affiliations:** 1 Fort Lauderdale Research and Education Center, University of Florida-Institute of Food and Agricultural Sciences, Fort Lauderdale, Florida, United States of America; 2 Plant Pathology Department, University of Nebraska, Lincoln, Nebraska, United States of America; 3 Hubbard Center for Genome Studies, University of New Hampshire, Durham, New Hampshire, United States of America; University of Waterloo, Canada

## Abstract

Large scale diversity patterns are well established for terrestrial macrobiota (e.g. plants and vertebrates), but not for microscopic organisms (e.g. nematodes). Due to small size, high abundance, and extensive dispersal, microbiota are assumed to exhibit cosmopolitan distributions with no biogeographical patterns. This assumption has been extrapolated from local spatial scale studies of a few taxonomic groups utilizing morphological approaches. Recent molecularly-based studies, however, suggest something quite opposite. Nematodes are the most abundant metazoans on earth, but their diversity patterns are largely unknown. We conducted a survey of nematode diversity within three vertical strata (soil, litter, and canopy) of rainforests at two contrasting latitudes in the North American meridian (temperate: the Olympic National Forest, WA, U.S.A and tropical: La Selva Biological Station, Costa Rica) using standardized sampling designs and sample processing protocols. To describe nematode diversity, we applied an ecometagenetic approach using 454 pyrosequencing. We observed that: 1) nematode communities were unique without even a single common species between the two rainforests, 2) nematode communities were unique among habitats in both rainforests, 3) total species richness was 300% more in the tropical than in the temperate rainforest, 4) 80% of the species in the temperate rainforest resided in the soil, whereas only 20% in the tropics, 5) more than 90% of identified species were novel. Overall, our data provided no support for cosmopolitanism at both local (habitats) and large (rainforests) spatial scales. In addition, our data indicated that biogeographical patterns typical of macrobiota also exist for microbiota.

## Introduction

Understanding spatial patterns of species diversity is important for setting priorities for conservation and monitoring and restoration programs. While large scale spatial patterns are well established for macroscopic eukaryotes (e.g. vertebrates and plants), for microscopic eukaryotes (e.g. nematode and mites) they remain greatly uncharacterized and underexplored. It has been assumed that microbiota exhibit cosmopolitan random distributions and lack biogeographical patterns [1] primarily due to their small size, astronomical abundance, and high dispersal rates [2]–[3]. However, the “everything is everywhere” (EisE) assumption has been extrapolated predominantly from studies at local spatial scales on protozoan taxa [4]–[5] using traditional morphological approaches. More recent molecular studies, however, provide strikingly contrasting evidence of very limited cosmopolitanism [6]–[9].

Nematode species richness is expected to exceed 1 million, but less than 4% is known to science [10]. This gap of knowledge is common to other eukaryotic microorganisms and generally results from the difficulty of applying traditional approaches (morphology and/or single organism PCR and sequencing) in species identification. Given that these taxa are major components of detrital foodwebs and play key roles as decomposers, predators, and parasites [11]–[12], it is critical to expand understanding of their biology and ecology. Knowledge of their spatial patterns is the first step to understanding their roles in ecosystem processes. As with protozoan species, the assertion of cosmopolitan distribution of nematode species can be traced back to several problems: 1) extrapolation from observations at small spatial scales, 2) use of morphological approaches [13] that prohibit identification at high taxonomic resolution, 3) bias towards agriculturally-relevant taxa and temperate regions, 4) processing of too few individuals from too few samples, and 5) absence of large spatial scale studies.

Ultrasequencing approaches offer an opportunity to accelerate the knowledge of the global biodiversity of microscopic eukaryotes by yielding more information faster and at lower cost than traditional approaches. Ecometagenetics has been successfully used to map prokaryotic diversity [14]. In this study, we used ecometagenetics to start mapping the diversity of microscopic metazoans. Specifically, we conducted a survey of nematode diversity (and other micro- and mesofauna) within three vertical strata or habitats (soil, litter, and canopy) of rainforests at two contrasting latitudes in the North American meridian (temperate at the Olympic National Forest, WA, U.S.A. and tropical at La Selva Biological Station, Costa Rica) using identical sampling designs and sample processing protocols [15]–[16]. Because two regions of the same gene (5′- and 3′- ends of the SSU rDNA) are currently in use in ecometagenetic analyses of marine nematodes [17], we tested this approach on samples from the temperate rainforest as well. Our work provides no support for cosmopolitan distribution of species and in fact points to the presence of patterns typical for macrobiota. However, the choice of sampled habitats and the primer sets may have a strong influence on diversity interpretation.

## Results

### Patterns for All Mesofauna

The total number of individual nematodes in the temperate rainforest varied from 8 to 222 per 100 ml within litter (L) and canopy (C) and from 960 to 2680 within the soil (S). In the tropical rainforest, the pattern was somewhat reversed, with lowest numbers (116 to 419) within canopy and soil, and highest (887 to 1490) within the litter. Temperate rainforest samples amplified from the 3′-end of the SSU diagnostic region generated a total of 42,023 high quality sequencing reads from which 17.2% were of nematode origin, 41.0% were identified as other microscopic eukaryota, 26.8% fell into a category of “environmental sample” (sequences with no taxonomic information in the NCBI database), and 15.0% were tagged as chimeric. Using the same diagnostic locus, these results contrasted with the tropical rainforest samples (a total of 171,861 high quality reads) for nematode and other eukaryote categories with 40% and 26%, respectively, but were similar for environmental samples (20%) and chimeras (14%) [16]. The temperate samples amplified on the 5′-end of the SSU diagnostic region, generated a total of 41,348 high quality reads, with only 4% of nematode origin, 41% of other eukaryotic origin, 25.9% as an environmental sample, and 29.3% as chimeras.

**Figure 1:**
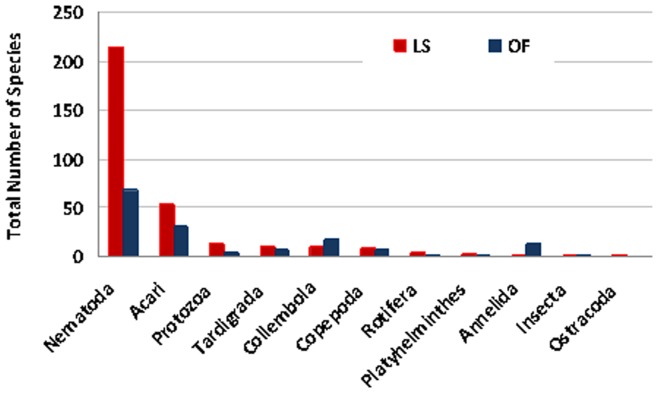
A comparison of total numbers of micro- and meio-faunal species between tropical (La Selva Biological Station, Costa Rica (LS)) and temperate (Olympic National Forest in WA, U.S.A. (OF)) rainforests from the 5′-end of the SSU diagnostic region.

Using the 3′-end generated datasets, a total of 157 micro- and mesofaunal putative species were observed across all habitats in the temperate rainforest as opposed to 323 putative species in the tropical rainforest [16] with nematodes as the most diverse and accounting for 44% (69 species) and 66% (214 species) of all the species, respectively. In both forests, mites were identified as the second most diverse group with their richness in the tropical rainforest almost twice as high as in the temperate (Figure 1). A similar pattern of decreasing richness from the tropical to the temperate rainforest was observed for all other groups except Annelids and Collembolans.

The total number of species recovered from the temperate rainforest depended on the choice of the diagnostic locus revealing that the overall species richness was lower by 30% when assessed with the use of the 5′-end versus the 3′-end locus. The bias was mostly directed against nematode taxa (Figure S1) with 49 (71%) fewer nematode species, but a similar decrease was observed for tardigrades.

### Patterns for Nematodes

**Figure 2:**
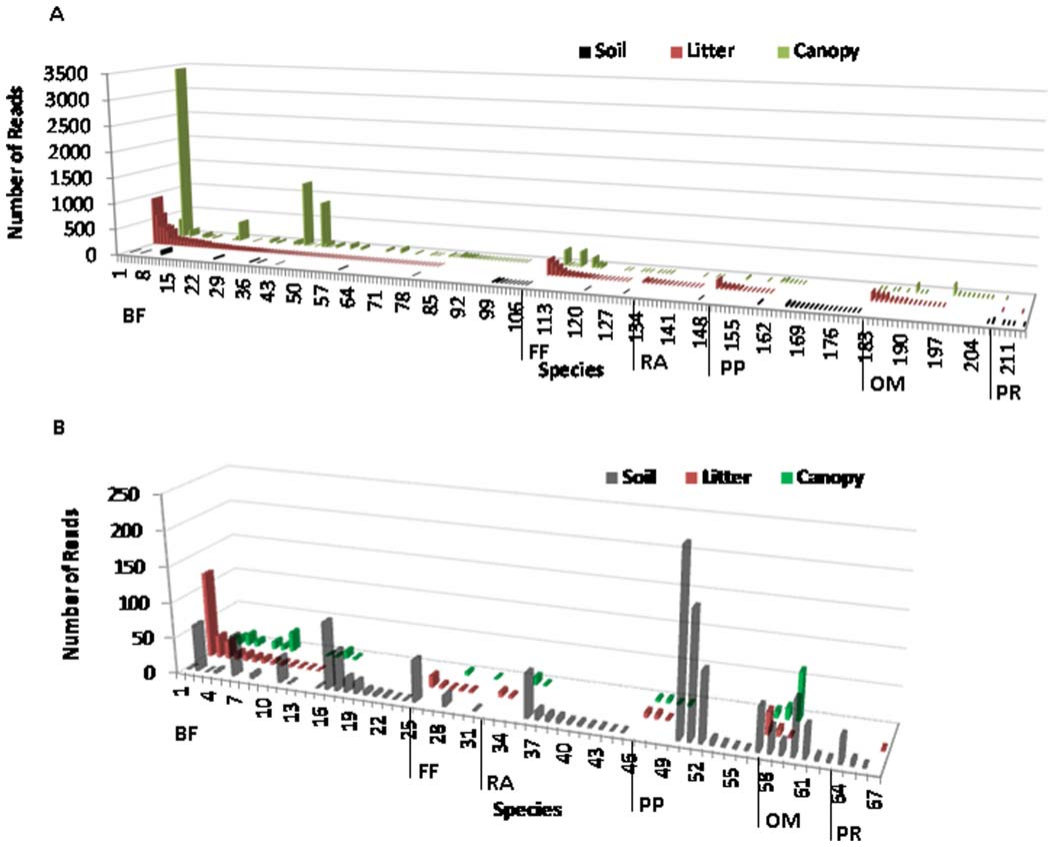
Average number of reads per species within Soil, Litter, and Canopy habitats. A) tropical rainforest at La Selva Biological Station in Costa Rica (LS), B) temperate rainforest at the Olympic National Forest in WA, U.S.A. (OF). Species are grouped into 6 trophic guilds: bacterial feeders (BF), fungal feeders (FF), root associates(RA), plant parasites (PP), omnivores (OM), and predators (PR) and reads are sorted from their highest to lowest numbers by the litter habitat within each trophic guild.

While the overall (across all habitats) nematode species richness and diversity were considerably higher in the tropical than in the temperate rainforest (Figure 2A,B, Figure S2A,B), the distribution of species within habitats was forest specific. More than 80% of all the species and nematode individuals in the temperate rainforest resided in the soil, whereas less than 20% in the tropics. While average richness, diversity and density were significantly higher (P<0.01) in La Selva than in the Olympic Forest within the litter and canopy habitats (Figure S2 A,B,C), the patterns were completely reversed within the soil habitat, with richness and abundance significantly (P<0.01) higher at the Olympic Forest than at the La Selva site (Figure S2 A,C).

The nematode assemblages were fundamentally different at two main levels: between the forests and among habitats within each forest. La Selva tropical rainforest and Olympic National Forest did not share even a single nematode species. Moreover, only 2% of La Selva species and 21% of the Olympic Forest species perfectly matched an existing sequence in the NCBI database. At the scale of each rainforest, nematode communities were very discrete with few shared species between habitats (Figure 2A,B). Only 6 (3%) out of 214 recognized nematode species at La Selva and 10 (15%) out of 69 in the Olympic Forest were shared among the soil, litter, and canopy (Figure S3). The shared species among habitats within the respective rainforests belonged to different taxonomic groups. At La Selva, they largely included bacterial feeders (Cephalobidae (2 spp), Rhabditidae (2 spp), Diplogastridae (1 spp)) and a plant parasite (*Xiphinema* (1 spp)) and at the Olympic Forest they included bacterial feeders (Plectidae (3 spp), Cephalobidae (2 spp), Teratocephalidae (2 spp)) and omnivores (Dorylaimidae (3 spp)). The unique composition was further magnified by the quantitative responses of the shared species. For instance, *Anaplectus* sp. 1 (Species #1 in Figure 2B) while completely dominating the litter community, fell into the “rare” species category within soil and canopy communities. Because the power of the statistical test was limited by patchy nematode distribution (high frequency of 0s), statistically significant differences were detected for only two tropical species (species #11 and #37 in Figure 2A).

### Nematode Diversity vs. Taxonomic Resolution

**Figure 3:**
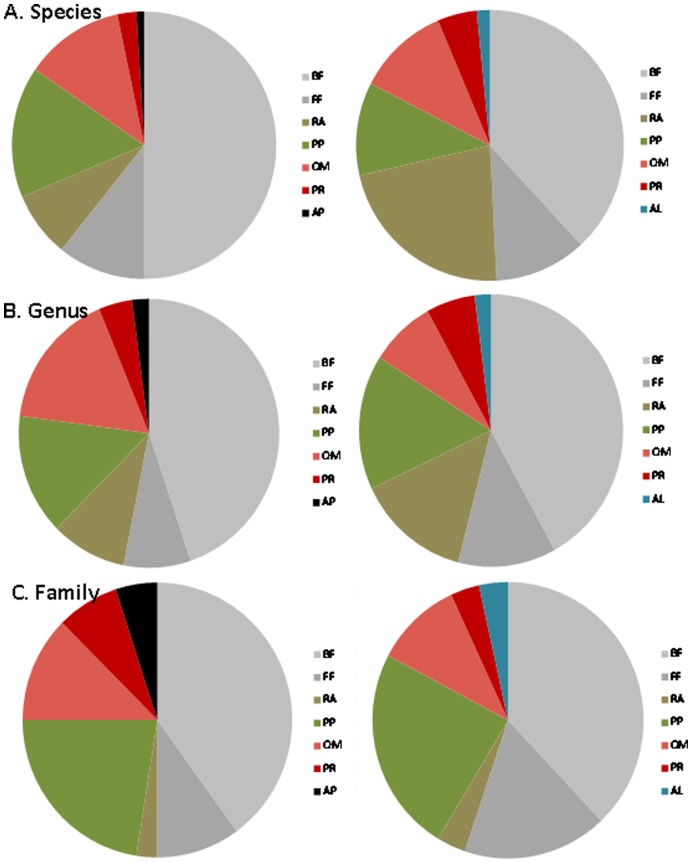
An overall (across all habitats) taxonomic richness of La Selva (left panel) and Olympic Forest (right panel) at different levels of taxonomic resolution. A) species, B) genus, and C) family. At each level of taxonomic resolution taxa were grouped by their feeding habit. BF  =  bacterial feeders, FF  =  fungal feeders, RA  =  root associates, PP  =  plant parasites, OM  =  omnivores, PR  =  predators, AP  =  animal parasites, AL  =  algivores.

The level of taxonomic resolution (e.g. species, genus, and family) affected the comparability of the two rainforests. At the species level, these two ecosystems were entirely different, with not even one species in common and 3.4 times higher richness at La Selva than the Olympic Forest. The trophic representation of the species was also different, with the largest differences for bacterial-feeding, root associated, and plant-parasitic taxa. Bacterial-feeding (50%) and plant-parasitic (16%) taxa contributed the most to the overall species richness at La Selva, versus bacterial-feeders (38%) and root-associates (22%) at the Olympic Forest (Figure 3A). At the genus level, the two ecosystems appeared more similar by sharing 22 taxa (44% of the total richness of the Olympic Forest, compared with 23% of total richness in La Selva), with richness only 1.9 times higher at the tropical than temperate rainforest. The proportional distribution of genera among trophic guilds converged for all but omnivorous taxa (Figure 3B). And finally at the family level, the two rainforests were nearly identical. The majority of taxa (86% of the total richness at the Olympic Forest and 62% at La Selva) were shared, and the proportional distribution of taxa among trophic guilds was similar (Figure 3C) as well as the estimates of richness were also similar (only 1.3 times higher at La Selva than the Olympic Forest). Despite this increasing similarity in nematode richness between the two rainforests as the level of taxonomic resolution decreased the communities remained fairly unique in the way different taxa contributed to the assemblages numerically even at the family level (Figure S4). Overall, bacterial-feeding taxa overwhelmingly dominated the tropical rainforest communities, while more even distribution was observed in the Olympic Forest. All shared taxa had very site specific numerical responses. For instance, while Rhabditidae and Cephalobidae (bacterial feeders) were the two most prevalent families in the tropical rainforest, Tylenchulidae (plant parasite) and Qudsianematidae (omnivore) dominated in the temperate forest. Breaking these communities into discrete micro-habitats (soil, litter, canopy) within each forest, further emphasized the uniqueness of each community just in terms of the shared taxa (Table 1). Out of 22 shared genera, only one (*Acrobeloides*) was present within all habitats in both forests in similar abundances and only two genera (*Pristionchus* and *Boleodorus*) were consistently found only within the soil environment. While at the family level the number of taxa displaying consistent responses across rainforests and habitats increased (6 out of 24), the majority of patterns were still unique.

**Table 1:** Relative abundance of shared taxa at the genus and family levels of taxonomic resolution.

Taxon	La Selva			Olympic Forest		
	Soil	Litter	Canopy	Soil	Litter	Canopy
**Genus (22)**						
Taxa common to both sites and all habitats						
(BF) *Acrobeloides*	* **2.1**	* **3.1**	* **1.3**	* **0.3**	* **5.2**	* **6.6**
Taxa common to both sites and almost all habitats						
(BF) *Oscheius*	22.2	**10.4**	**38.5**		**0.3**	**0.9**
(BF) *Plectus*		**20.4**	**6.2**	4.7	**9.5**	**4.6**
(BF) *Teratocephalus*		**0.1**	**0.9**	4.1	**2.8**	**5.5**
(FF) *Tylencholaimus*	**0.3**	**5.9**	0.2	**4.1**	**5.0**	
(PP) *Xiphinema*	10.8	**0.1**	**0.3**		**2.0**	**2.0**
Taxa common to both sites but only few habitats						
(BF) *Anaplectus*		**2.0**		0.1	**36.6**	2.6
(BF) *Geomonhystera*		1.7	**0.5**			**4.8**
(BF) *Howardula*	* **1.8**	1.6		* **0.3**		
(BF) *Pristionchus*	* **0.4**			* **0.5**		
(BF) *Rhabditis*	6.6	**0.4**			**3.0**	
(BF) *Trypilina*	1.4	**4.1**			**1.1**	
(FF) *Aphelenchoides*		**8.4**	8.5	1.1	**2.2**	
(FF) *Diptherophora*	0.2				0.8	
(RA) *Aglenchus*		0.1		0.3		
(RA) *Boleodorus*	* **0.2**			* **2.2**		
(RA) *Filenchus*		**0.3**	0.1	4.4	**2.2**	
(PP) *Anguina*		0.1				0.9
(PP) *Meloidogyne*	0.9		**0.1**		0.7	**0.7**
(OM) *Mesodorylaimus*		5.7	0.4	0.3		
(PR) *Mylonchulus*	**0.7**	0.1	0.1	**0.6**		
Total %	47.5	64.2	56.9	20.9	71.5	28.4
**Family (24)**						
Taxa common to both sites and all habitats						
(BF) Cephalobidae	* **5.4**	* **10.9**	* **32.7**	* **0.3**	* **12.8**	* **13.6**
(RA) Tylenchidae	* **0.3**	* **3.0**	* **0.3**	* **7.7**	* **3.4**	* **5.9**
Taxa common to both sites and almost all habitats						
(BF) Monhysteridae		**1.8**	**0.5**	2.5	**0.7**	**4.8**
(BF) Plectidae		**22.3**	**6.3**	5.4	**48.4**	**20.8**
(BF) Rhabditidae	28.8	**12.9**	**38.7**		**3.3**	**0.9**
(BF) Teratocephalidae		**0.5**	**1.6**	4.1	**2.9**	**5.5**
(BF) Trypilidae	**1.4**	**4.2**	0.5	**1.2**	**1.1**	
(FF) Tylencholaimidae	0.3	**5.9**	0.2	4.1	**5.0**	
(PP) Belonolaimidae	**1.6**	**0.2**		**0.2**	**2.1**	1.8
(PP) Heteroderidae	**0.9**		**0.0**	**0.1**	0.7	**0.7**
(PP) Longidoridae	10.8	**0.0**	**0.3**		**2.0**	**2.0**
(OM) Quadsianematidae		**3.0**	**1.6**	13.2	**13.0**	**40.3**
Taxa common to both sites but only few habitats						
(BF) Allantonematidae	**1.9**	1.6		**0.3**		
(BF) Diplogastridae	**4.2**	0.3	0.5	**0.5**		
(BF) Mermithidae	* **4.2**			* **10.6**		
(FF) Aphelenchoididae		**10.2**	10.1	1.1	**2.3**	
(FF) Diptherophoridae	0.2				0.8	
(FF) Leptonchidae		0.7		0.2		
(PP) Anguinidae		6.6	1.1			0.9
(PP) Criconematidae	* **23.5**			* **0.3**		
(PP) Ecphyadophoridae	* **0.2**			* **0.6**		
(PP) Tylenchulidae	* **4.6**			* **38.6**		
(OM) Dorylaimidae		8.0	0.8	0.7		
(PR) Mononchidae	**2.2**	0.0	0.0	**3.3**		
Total%	90.1	91.9	95.2	95.1	98.5	96.4

Bold indicates taxa common to specific habitats.

* indicates taxa common to both sites and consistent habitats. BF  =  bacterial feeders, FF  =  fungal feeders, RA  =  root associates, PP  =  plant parasites, OM  =  omnivores, PR  = predators.

### Nematode Diversity vs. Diagnostic Locus

**Figure 4:**
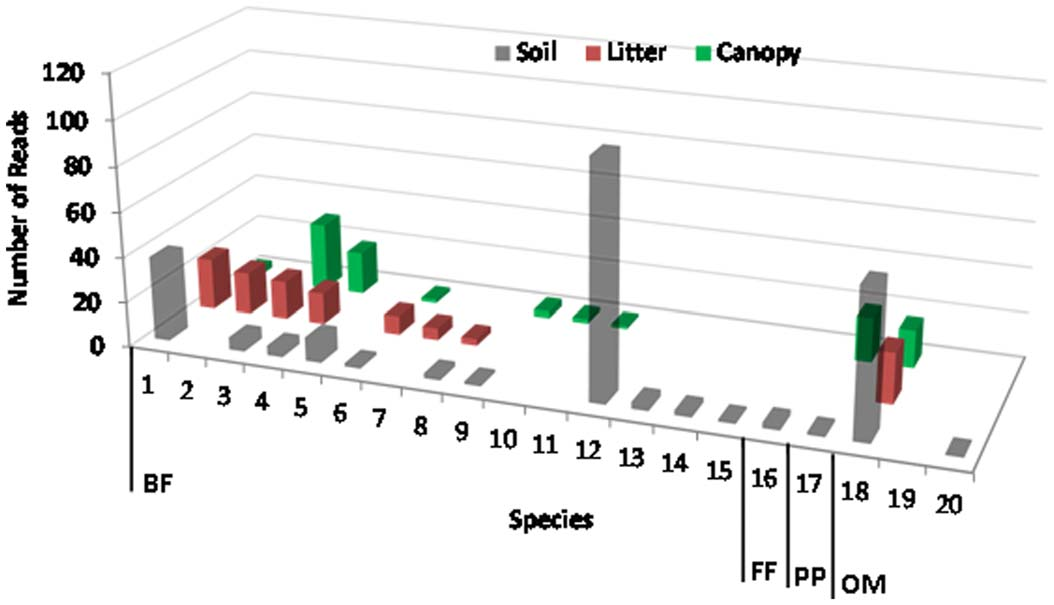
Average number of reads per species within Soil, Litter, and Canopy habitats in the temperate rainforest at the Olympic National Forest in WA, U.S.A. generated from the use of the 5′-end diagnostic locus. For a comparison to the results from the use of 3′-end see Figure 2B. Species are grouped into trophic guilds: bacterial feeders (BF), fungal feeders (FF), plant parasites (PP), omnivores (OM) and reads are sorted from their highest to lowest numbers by the litter habitat within each trophic guild.

**Figure 5:**
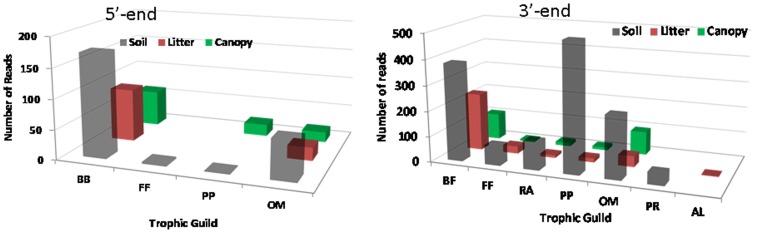
A nematode community composition at the trophic guild level depending on the choice of the diagnostic locus (5′-end or 3′-end of the SSU rDNA).

In comparison to species richness estimated using the 3′-end diagnostic locus (total species  = 63) (Figure 2B), the 5′-end underestimated species richness by 60% (total species = 20) (Figure 4). For bacterial feeders, the identified species were largely parallel. For fungal-feeding, plant-parasitic, and root-associated taxa, however, species richness and abundance estimates were so incongruent that even at the trophic guild level, the 3′-end and 5′-end recovered nematode communities had very little in common (Figure 5). In order to explain this discrepancy, we investigated priming regions of all identified species for which full length SSU sequences have been published. A total of 47 full length SSU sequences (17 out of 20 5′-end identified species, and 30 out of 62 3′-end identified species) were downloaded from the NCBI and aligned in MEGA5 using default parameters. The 3′-end priming regions (both forward and reverse) were 100% identical across all 47 taxa. In contrast, the 5′-end priming sites had 1–2 bp differences, and the great majority of the mismatches were observed among fungal-feeding, plant-parasitic, and root-associated tylenchids.

## Discussion

Elucidation of spatial patterns of species diversity is critical. Not only does it help us with establishing the theoretical mechanisms of diversity and trajectories of evolution, but it also helps in setting practical priorities for conservation, monitoring, and restoration efforts of ecosystems and their functions. Given that the great majority of the biodiversity resides not with macroscopic, but with microscopic taxa, it is surprising that microbial biogeography still lacks a map. Also, given that microscopic taxa play key roles in ecosystem functioning through decomposition and nutrient mineralization processes, it is surprising that we still do not know the answers to most of the questions of who, how many, where, and how. Lastly, given that the 21^st^ century molecular advances have revolutionized all aspect of biological sciences, it is surprising that the science of microscopic biogeography is still largely resigned to early 20^th^ century hypotheses [1], [18]. Massive sequencing technologies now allow for large-scale, standardized, and effective (time and cost) biodiversity assessments, challenging the concept of EisE with meaningful data.

Here, we used pyrosequencing to draw insights on spatial diversity patterns of nematodes (but also by other similarly sized invertebrates). Even with the relatively low number of samples, the emerging patterns unambiguously contradicted the EisE hypothesis on several levels. At the large spatial scale, not even a single species could be classified as widely distributed. The nematode communities of the two rainforests were of completely different species compositions. Very few common species among habitats within each rainforest further exemplified the lack of unrestricted distributions of nematode species even at the local scale. Moreover, the low number of perfect matches to sequences (presumably from all over the world) within public databases, could be treated as further evidence against EisE. The results of our study are in line with the results of other recent studies applying molecular approaches. Wu et al. [9] examined environmental SSU rDNA sequences of soil animals from 11 locations from different biomes at various latitudes. Just as in our study, they found that 95.8% of all (2,259) OTUs (assembled at 99% similarity that is considered operationally equivalent to a species level) were present at just a single location and no OTU was common to all locations. In the analysis of 26 *Caenorhabditis* species using 11 genes, Kiontke et al. [19] found that only *C. briggsae* was truly cosmopolitan. All other species were confined to a very specific geographical area (e.g. East Asia, West Africa, or South India). Using the cox1 gene, Robeson et al. [8] looked at the diversity patterns of bdelloid rotifers, a taxon most likely to exhibit cosmopolitan distributions (abundant, anhydrobiotic, asexual, not overly specious) [20]. They observed autocorrelation at the local scale (up to 133 m), but beyond that distance (up to 10,000 km) communities were extremely dissimilar predominantly due to the presence of previously unrecognized cryptic species. This recently emerging pattern of highly endemic rather than cosmopolitan taxa not only for microscopic eukaryotes but prokaryotes as well [21] can be largely attributed to the use of molecular methods that are much more sensitive in species recognition than methods based solely on morphology. Due to a limited number and easy to observe morphological characters in microscopic organisms, what appears as a few globally distributed morphological species, often turns out as numerous phylogenetic species that are relatively site specific [22]. In the example of the *Caenorhabditis* study [19], morphology can be used to assign species to the major supergroups (e.g. *Elegans* and *Drosophila*) but within each group some species are considered sibling taxa and look identical. More importantly, almost none of the morphological characters analyzed had an unambiguous distribution when superimposed on the phylogeny reconstructed from molecular data indicating numerous conflicts between morphology and molecular characters.

The uniqueness of nematode communities to rainforests and habitat types within each rainforest provided us with evidence corroborating the idea that micro-invertebrates are not very different in their spatial patterns from macroorganisms. However, evidence that relates to the species numbers along a latitudinal gradient is even more critical. Nematode species, unlike those of macrotaxa, have been predicted to exhibit a peak of their diversity in temperate regions rather than at the equator. This peak is potentially artifactual for the following three reasons: 1) oversampling of temperate and undersampling of tropical regions; 2) dependence on morphology of low taxonomic resolution in nematode diagnostics; and 3) the use of small scales and disconnected studies with highly variable methodologies for inference about the large scale distribution patterns. Powers et al. [15] and Porazinska et al. [16] provided preliminary support for compliance with latitudinal gradients in detailed studies of tropical rainforest nematode diversity. Whether using traditional molecular (single organism PCR/Sanger sequencing) or novel (pyrosequencing) tools, they reported that the overall nematode species richness was high (167 and 214 observed species, respectively), potentially contradicting current dogma. Boag and Yeates [13] reviewed 134 studies from different ecosystems around the world and noted temperate broadleaf forests with an average of 67 nematode species (morphologically identified) as the most diverse. Tropical rainforest lagged far behind with an average of only 33 species. Since sampling strategies most likely were limited to soil, their results are remarkably consistent with our data for the soil habitat. But Powers et al. [15] and Porazinska et al. [16] showed that the diversity in the tropical rainforest reached from the belowground into the canopy stratum, suggesting that estimates of terrestrial nematode diversity exclusively based on surveys of soil habitats were inadequate. They hypothesized that in humid tropical ecosystems, suitable temperature and moisture conditions are not restricted to the confines of the soil environment but extend into the aboveground and that this vertical distribution of nematode habitats may become compressed with increasing latitude restricting nematode presence to the conventional soil environment. The most logical next step to test this hypothesis was to replicate the sampling design and sample processing protocols in a temperate rainforest, an ecosystem most equivalent in structure and function to the tropical rainforest. Both ecosystems receive ∼4 m of rain per year, and extensive growth of plant-life (e.g. plants, ferns, mosses) expands from the floor into the tree trunks and branches. With this more appropriate comparison, not only overall nematode richness, but also richness of other meiofaunal taxonomic groups followed the latitudinal gradient with richness 300% higher in the tropical than temperate rainforest. However, as we hypothesized, the distribution of species among habitats was of opposing patterns. Eighty percent of the species resided in the soil in the temperate rainforest, whereas only 20% in the tropics. If we had only sampled the soil environment, 80% of the tropical nematode diversity would have gone undetected, corroborating yet again the presumed diversity peak in the temperate region. This result is significant because it illustrates how unintentionally biased sampling designs can ultimately skew our conclusions. Nematodes, although traditionally conceptualized as soil organisms, are really aquatic [23] and they will thrive wherever a film of water can support them. In grasslands or desserts, they are most likely to be confined to the soil environment, but in more three–dimensional ecosystems with higher aboveground structure and complexity, their habitats can be extended above the soil layer. In the case of our study, the reverse pattern of diversity among habitats was likely driven by soil and litter properties. With no hard data in hand, we could only speculate that highly organic soils in the temperate rainforest did what a thick and diverse litter layer did in the tropical rainforest: provided ample food resources to support a diverse nematode community. In contrast, limited food resources within poor soils (minimal organic matter) in the tropics [24] and scant litter layer (often overgrown by a single moss species) in the temperate rainforest restricted the nematode diversity in the respective habitats. Again, temperature is likely the main underlying factor setting differences in the rates of soil organic matter decomposition and litter layer accumulation, and consequently in diversity patterns within specific habitats. Overall nematode diversity, however, was probably influenced by a combination of temperature and plant diversity, but remains to be tested.

This strict adherence to soil sampling has possibly contributed to the lack of observance of latitudinal patterns in the most recent study of worldwide distribution and diversity of soil animals [9]. For instance, if sampled aboveground, the Costa Rican tropical rainforest (the same one we sampled) and the Peruvian tropical rainforest would be probably more on par with the Kenyan grassland, the most diverse ecosystem right at the equator. We often strive for standardized methodologies, but it is clear that identical sampling across structurally divergent ecosystems does not equate to appropriate sampling strategy. Kiontke et al. [19] elegantly showed the slow rate of discovery (22 species over ∼120 years) of a presumably soil inhabiting *Caenorhabditis* species, but once it was realized it is a rotting fruit inhabitant, the rate increased with 16 new species just within the last 6 years. Clearly, describing diversity will require expanding our repertoire of sampling strategies.

As much as sampling strategies reflecting an ecosystem’s complexity and structure will play a role in adequate assessments of meiofaunal diversity, the diagnostic loci selected for the assessments will be just as important. For the assessment of the diversity within the Olympic Forest, we expanded our previous work to use two loci, 5′- and 3′- end of the SSU [25]. While the 5′-part of the SSU might be more desirable because of higher sequence divergence and resolving power [25], it turned out to be inferior to the 3′-part as the less conserved priming region failed to amplify many Tylenchina known as fungal-feeders and plant-parasites/associates [26]. A bias like this not only misrepresents the diversity, but also distorts inferences about ecosystem functioning by omitting whole groups of taxa. Clearly, a locus with a conserved and stable priming region for amplifying across all taxonomic groups, even if less resolving, is more appropriate. In a study of the diversity patterns of marine nematodes, Bik et al. [17] used the same two regions without any observable biases. However, tylenchids constitute a group of nematodes derived from marine ancestors that invaded terrestrial habitats [27], and an apparent divergence within the priming region makes the 5′ section of the SSU rDNA sub-optimal for terrestrial nematode diversity studies. An alternative to 5′ and 3′ sections of the SSU rDNA, a mid section was used in the worldwide study of soil animals [9]. Because the estimates of richness for the Cost Rican rainforest were somewhat lower than ours, we briefly investigated the conservation of their primers using the same dataset of the 47 full SSU sequences used to compare 5′- vs. 3′- rDNA diagnostic loci for our temperate rainforest study. As suspected, we observed 1–3 bp mismatches for 60% of nematodes species across all phylogenetic clades. In contrast, the priming regions of the 3′-end diagnostic locus that we used in both rainforest studies were extensively tested (∼2,000 NCBI SSU eukaryotic sequences covering all phyla) and reported to be uniquely conserved, particularly within Nematoda [28]. While the development and availability of alternative primers to diagnostic regions is urgently needed, these alternative primers must be thoroughly vetted to avoid gross taxonomic biases.

As mentioned above, methodological biases not only can affect the perceptions of the general diversity patterns, but most importantly can affect the perceptions of a wide variety of ecological concepts ranging from community composition, to roles of specific species in ecosystem functioning, to species redundancy, or relationships between diversity in the belowground and the aboveground. As shown in our study, the composition and structure of nematode communities differed not only between the rainforests but also at the local scale of individual habitats. Because of the very low overlap of species among habitats and between the forests, it appears that the roles that species play within each community are very narrow and are defined by the specific conditions of each environment. It also suggests that species, even when considered functional equivalents (e.g. bacterial-feeders), may not be functionally identical and thus not, as often assumed, redundant. In the tropical rainforest, for example, a different set of species were part of the decomposition process. *Oscheius* sp. A, *Cephalobus* sp. A, B, C (BF) and *Aphelenchoides* sp. A, B, C (FF) appeared to be the key players in the canopy, while *Plectus parvus*, *Oscheius* sp. A, *Myolaimus* sp. A (BF) and *Aphelenchoides* sp. A, D, and *Tylencholaimus* sp. A (FF) were the key players within the litter. None of the above species, however, participated in the process within the soil environment with *Oscheius* sp. B (BF) filling this role. As we move to a temperate rainforest, the pattern was similar such that three completely new sets of species were most significant. Importantly, the guilds of species were not merely simple, closely-related replacements, but instead were phylogenetically diverged lineages that have evolved over potentially long period of time to fill these roles.

With new molecular approaches, we are just scratching the surface of the ecology of microscopic communities and their significance to ecosystem functioning. A temporal component in our study would probably further highlight the specificity and the importance of each species in different places at different times indicating that dominance and rarity can be fluid. Isbell et al. [29] studied plant species in 17 biodiversity experiments and while species generally appeared redundant when considered under one set of environmental conditions in the context of one ecosystem function, 85% of species were needed to maintain multiple functions at multiple places and multiple times. This same pattern is likely to emerge from microscopic communities. However, the reliance on adequate sampling and diagnostic loci cannot be overemphasized. The inverse relationship between soil organisms and aboveground plant diversity suggested by Wu et al. [9] was a likely artifact of problems associated with both sampling error and the selective nature of their primers. It is no surprise to observe low diversity of soil communities in high diversity conservation areas such as Costa Rican rainforest, where 80% of meiofaunal species reside not in the soil but in the aboveground. From our own study, *Caenorhabditis briggsae*, a rare species with known widespread distribution [19], was expected to be found in both forests. While it was detected in a single soil sample at La Selva, it was entirely absent from the Olympic Forest. Knowing now that rotting fruit, not soil, is the preferred habitat, its absence in most of our samples is not an enigma. These examples illustrate that ecological concepts, such as relationships between belowground and aboveground diversity, have to take into account the structure and complexity of a studied ecosystem.

Another notable difference between the rainforests was that in contrast to the temperate region, almost every nematode genus in the tropical system (particularly within litter and canopy) was represented by several (possibly closely-related) species potentially pointing out, as predicted, to a higher resilience of the tropics than temperate regions to environmental disturbances. The traditional use of morphological characters would likely fail to distinguish these subtle differences and ultimately result in underestimates of species richness as well as an inability to recognize the uniqueness of each community. High-throughput sequencing allows us to execute diversity assessments faster and cheaper, but most importantly to examine the diversity of microscopic organisms at the species level of resolution. Only at the species level, can we appreciate the commonality of endemism vs. rarity of cosmopolitanism. Predictably, as the resolution declines, communities become more and more similar and the pattern of cosmopolitanism falsely appears. Similar recent observations were made for nematode, rotifer, tardigrade, and fungal taxa [8], [19], [22], [30]–[31] where cosmopolitan “phenotypic species” were actually phylogenetic species complexes, and when finally individually recognized, they showed significant endemism. While the 3′-part of SSU performed reasonably well to uncover species diversity in our study, it undoubtedly underestimated the true extent of endemism. The SSU DNA has often been shown to offer limited resolution for closely related/cryptic species [32]. As we develop primers of greater taxonomic discrimination, e.g. COI primers [33], and use them in parallel to SSU, we are likely to reinforce the main conclusions of this study.

## Methods

### Sampling and Extraction

In order to be able to make a direct comparison to our results from Costa Rican tropical rainforest (all necessary permits were obtained to this field study, see acknowledgments), we followed similar protocols for nematode sampling and extractions, DNA extraction, amplification, and sequencing, as well as sequencing tag processing. Explicit details can be found in Porazinska et al. [16]. Briefly, in September 2010 we collected soil, litter and canopy samples from a temperate rainforest at the Olympic National Forest near the Lake Quinault, WA. Samples were collected at 4 locations (replicates) separated from each other by approximately 100 meters. Within each location, not larger than 1500 m^2^,4 random canopy trees and 4 random understory trees were selected as sampling points (a total of 8/replicate). One soil (15 cm depth) and one litter (any organic material overlying the soil) sample was collected from a 15 cm×15 cm area within 1–2 m away from the canopy and the understory trees. All eight samples were combined to make up one composite soil and one composite litter sample per each sampling location. A canopy sample was made up of epiphytic material (e.g. lichen, moss, algae) present on the surface of stems of canopy and understory trees. Each tree was sampled at three vertical points (base of the tree, 1 m and 2 m above the soil) from a 15×15-cm area. A total of 24 subsamples (3 vertical points×8 trees) were pooled together to form one composite canopy sample per each sampling location. No specific permits were required for this field study. Samples were stored in a cooler and transported to Oregon State University and USGS in Corvallis, OR for immediate processing.

To ensure maximum recovery of nematodes (and other similarly sized fauna) from different habitats (non-buoyant soil vs. buoyant organic and plant material), we used two different extraction methodologies. A hundred ml of soil subsamples (equivalent of ∼70–80 g) was processed using sugar flotation and centrifugation (based on passive separation due to density differences of nematodes and soil particles) [34], and 100 ml of litter and canopy material (equivalent of 15–30 g) were extracted using Baermann funnels (based on active migration of nematodes) [35]. Prior to being placed in funnels, litter and canopy material was first cut into smaller pieces, mixed, and 100 ml subsamples were chopped in a blender in 150 mL of deionized water for 10 s. Nematodes were collected after 48-hr. All extracted nematodes were counted immediately for abundance at the trophic group level under an inverted microscope, reduced to 0.5 ml, transferred into ZR BashingBead Lysis Tubes (Zymo Research Corp, Santa Ana, CA) and transported to the University of Florida for DNA processing.

### DNA Extraction, Amplification, and Sequencing

ZR Tubes were processed at maximum speed for 2 minutes on a Mini-BeadBeater (BioSpec Products, Inc. Bartlesville, OK). Genomic DNA was extracted using ZR Soil Microbe DNA kit according to the manufacturer’s protocol. Similarly to the tropical samples, eluted DNA was used as a PCR template for amplification of a ∼400 bp diagnostic region within 3′-part of SSU rDNA: NF1/18Sr2b [28], [36]. In addition to 3′-part of the SSU, a diagnostic region within the 5′-part of SSU rDNA: F04/R22 [37] was amplified using the same DNA template (tropical samples 3′-part of SSU, temperate samples 3′- and 5′-part of SSU). PCR amplifications were performed following protocols described elsewhere [17] using MID-tagged (10 nucleotides) fusion primers as opposed to 2 nucleotide MID tags that were used for tropical samples [16]. All temperate rainforest metagenetic SSU samples were sequenced on two (to accommodate two diagnostic regions) Genome Sequences Titanium (Roche/454 Life Sciences) half-plates (along with other samples) at the Interdisciplinary Center for Biotechnology Research (ICBR) at the University of Florida, Gainesville, FL (tropical samples were run on an earlier version GS FLX). Earlier experiments with artificially assembled nematode communities established that the use of a single PCR reaction and a single emulsion PCR and pyrosequencing run were sufficient for both qualitative and quantitative analysis of the nematode community composition and structure [36].

### Metagenetic Sequence Processing

Generated sequences were processed using an OCTUPUS (**O**perational **C**lustered **T**axonomic **U**nits for **P**arallel-tagged **U**ltra **S**equencing) bioinformatics pipeline [25] that has been benchmarked against other pipelines used for prokaryotes [17], [38]. OCTUPUS scanned sequences for quality using Lucy-trim with default parameters [39] and screened them for a minimum length of 200 bp, and then binned them by their MID tags. Sequences were then clustered to OCTUs (Operational Clustered Taxonomic Units) at 99% similarity using MEGABLAST [40] and MUSCLE to generate a list of “fixed” OCTUs (an OCTU consensus achieved when an addition of a sequencing read to an OCTU group does not result anymore in a change of the OCTU consensus). The level of 99% within OCTU similarity was determined to be the most appropriate for recognizing the relationship between OCTUs and putative species [41]. Fixed OCTUs were blast-matched [42] against the NCBI database, expanded by the nematode reference sequences from our control experiments [28], [36] and nematode reference sequences from Costa Rica [15]. The reference sequences were generated by single nematode PCR followed by Sanger sequencing. The similarity cut-off for identifying OCTUs was set to no less than 90%. All OCTUs were analyzed for the presence of putative chimeras using a frequency and length dependant algorithm incorporated into the OCTUPUS pipeline. Chimera tagging is reference database independent and instead compares OCTUs against each other. It is based on the assumption (as in other algorithms like Perseus or UCHIME) that chimeric sequences are less frequent than their parental sequences. OCTU sequences are compared along their total lengths. A chimeric sequence is detected when two sequences initially match at high identity on the 5′-end but differ greatly on the 3′-end resulting in incomplete length match. Based on the analysis of control datasets from artificially-assembled nematode communities [43], all OCTUs with incomplete length match of ≥10 bp were flagged as chimeric. Consequently, all OCTUs flagged as chimeric were removed from the analysis of nematode OCTUs.

### Analyses

Because OCTUs generated from SSU rDNA by ultrasequencing are not equivalent to species, OCTUs per se were not used for the analysis of biodiversity. Instead, all high quality nonchimeric OCTUs were linked back to putative species by using Head-Tail patterns identified and described from artificially assembled nematode communities [41]. Briefly, in metagenetic datasets generated from SSU rDNA by ultrasequencing, a single species is usually represented by series of OCTUs and each OCTU by multiple sequencing reads [41]. The most frequent OCTU of a species, Head, is characterized by the highest bioinformatics scores resulting from blast-matching it to the database reference sequence, and less abundant OCTUs, Tail, with slightly variant sequencing reads by lower scores to the same matching reference sequence. When sorted by the scores, predictable Head-Tail patterns emerge. The presence of two-three Heads of similar scores and read frequency, on the other hand, indicates the presence of closely related or cryptic species [41]. To infer about quantitative relationships, all reads within each OCTU (Head and Tail) linked to a putative species were summed up to generate abundance per species per sample. Nematode species were grouped into less resolved taxonomic groupings such as genera and families, but also into functional guilds (bacterial-feeders, fungal-feeders, omnivores, plant-parasites, predators, root associates, and animal parasites) following Yeates *et al*. [26]. EstimateS [44] was used to compute species richness (expected and total predicted) [45], and diversity (Shannon-Weaver) [46]. For richness and diversity estimates within habitats, input data into EstimateS consisted of a matrix of the list of species and their abundances per every replicate within each habitat (e.g. N = 4 for soil in the tropical rainforest). For total richness and diversity across all habitats within each rainforest, all species in all habitats in all replicates were used (e.g. N = 12 for tropical rainforest). EstimateS derived richness and diversity for each sample were then averaged across each habitat (e.g. N = 4 for soil in the Tropical rainforest) or across the entire rainforest (N = 12 for the tropical rainforest). Because of the presence of no reads for many species and very high read number variation, calculations in EstimateS were performed on transformed/normalized data (numbers of sequencing reads per each putative species were transformed into numbers of nematode individuals per putative species using guidelines from control experiments with artificially-assembled nematode communities) [36]. Two-way analysis of variance (ANOVA) was used to detect statistical differences between rainforests and among habitats in species richness, diversity, and the number of real individuals (density). For shared species among habitats within each rainforest (6 in tropical and 10 in temperate), one-way analysis of variance was used to detect differences in abundance. Because of high variance, abundance was log(x+1) transformed prior to analysis. The StatistiXL data analysis package as an Add-In to Excel 2007 was used for both cluster and ANOVA analyses. From the 12 metagenetic temperate rainforest samples, three samples (L1, C2, C4) that were amplified on the 3′-end of the SSU and 2 samples (L3 and C3) that were amplified on the 5′-end of the SSU generated no or only a few sequencing reads and were therefore removed from analyses.

While our sampling, extraction, and metagenetics methodologies are fine-tuned for nematode taxa, they are not selective against other microscopic eukaryotes (e.g. mites, tardigrades, springtails). Because ∼50% of the metagenetic data consisted of non-nematode sequences, they are presented in this paper as well, although these could be subject to sampling, extraction, and amplification biases. All methods and analyses, including bioinformatics, were the same as for nematodes.

### Data Accessibility

Raw 454 read data along with metadata describing specific primers and MID-tags have been deposited at the Short Read Archive at the NCBI under the following submission numbers: Study: SRSPO14451, Sample 1: SRS350224 (diagnostic locus covering the 3′-part of the 18S) and Sample 2: SRS350225 (diagnostic locus covering the 5′-part of the 18S).

## Supporting Information

Figure S1 **A comparison of total numbers of micro- and meio-faunal species between 3′- and 5′- end diagnostic loci in the temperate (Olympic National Forest in WA, U.S.A) rainforest.**
(XCF)

Figure S2 **Average diversity and abundance within soil, litter and canopy habitats and across all habitats (Total) in the tropical rainforest at La Selva Biological Station in Costa Rica (LS), and the temperate rainforest at the Olympic National Forest in WA, U.S.A. (OF).** A) Richness (number of species), B) diversity (Shannon), and C) Abundance (number of nematode individuals per 100 cc). Bars indicate standard errors.
(TIF)

Figure S3 **Percent of shared species among habitats (soil  =  S, litter  =  L, and canopy  =  C) in the tropical rainforest at La Selva Biological Station in Costa Rica (LS), and the temperate rainforest at the Olympic National Forest in WA, U.S.A. (OF).**
(TIF)

Figure S4 **A comparison of overall nematode assemblages between temperate (Olympic Forest, OF) and tropical (La Selva, LS) rainforests at the family level of taxonomic resolution.** Families were grouped by their trophic guilds and sorted within each guild by their proportionate representation (highest to lowest within LS). BF  =  bacterial feeders, FF  =  fungal feeders, RA  =  root associates, PP  =  plant parasites, OM  =  omnivores, PR  =  predators, AP  =  animal parasites, AL  =  algivores.
(TIF)
