# Supplementary figures and images for: Nematode Spatial and Ecological Patterns from Tropical and Temperate Rainforests
Source: PLoS One. 2012 Sep 11;7(9):e44641. doi: 10.1371/journal.pone.0044641 (PMC3439412; doi:10.1371/journal.pone.0044641)

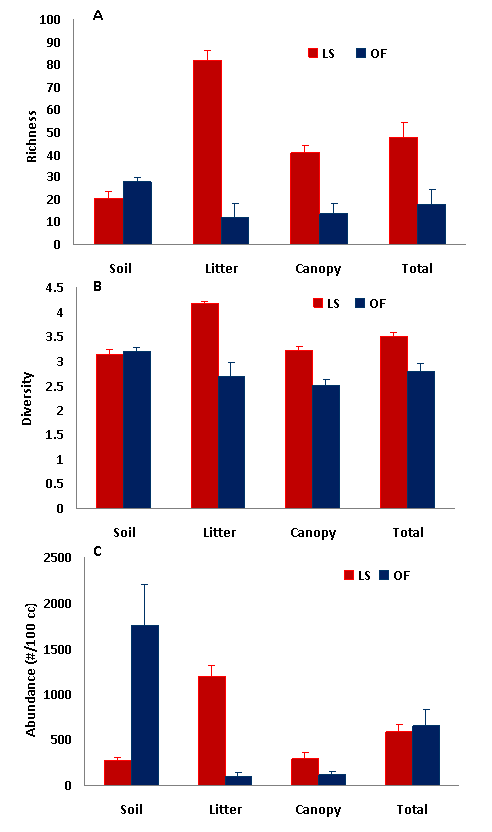

Supplement: Figure S2 — Average diversity and abundance within soil, litter and canopy habitats and across all habitats (Total) in the tropical rainforest at La Selva Biological Station in Costa Rica (LS), and the temperate rainforest at the Olympic National Forest in WA, U.S.A. (OF). A) Richness (number of species), B) diversity (Shannon), and C) Abundance (number of nematode individuals per 100 cc). Bars indicate standard errors. (TIF) [file pone.0044641.s002.tif]

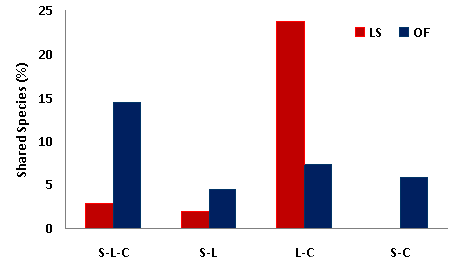

Supplement: Figure S3 — Percent of shared species among habitats (soil = S, litter = L, and canopy = C) in the tropical rainforest at La Selva Biological Station in Costa Rica (LS), and the temperate rainforest at the Olympic National Forest in WA, U.S.A. (OF). (TIF) [file pone.0044641.s003.tif]

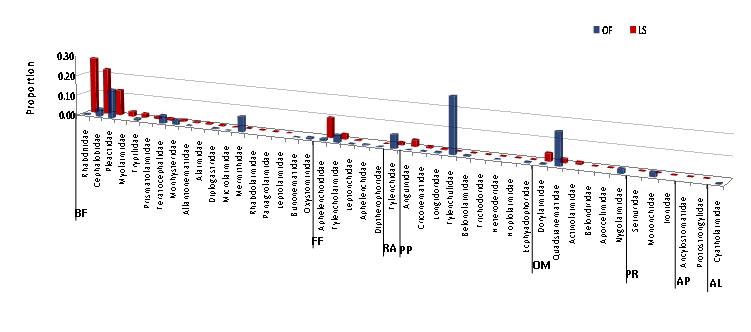

Supplement: Figure S4 — A comparison of overall nematode assemblages between temperate (Olympic Forest, OF) and tropical (La Selva, LS) rainforests at the family level of taxonomic resolution. Families were grouped by their trophic guilds and sorted within each guild by their proportionate representation (highest to lowest within LS). BF = bacterial feeders, FF = fungal feeders, RA = root associates, PP = plant parasites, OM = omnivores, PR = predators, AP = animal parasites, AL = algivores. (TIF) [file pone.0044641.s004.tif]
